# Necrotising fasciitis secondary to a perforated hepatic flexure tumour — A case report

**DOI:** 10.1016/j.ijscr.2023.108619

**Published:** 2023-08-04

**Authors:** Sarada Ganesan

**Affiliations:** Department of Surgery, Wollongong Hospital, Wollongong, New South Wales, Australia

**Keywords:** Necrotising fasciitis, Perforated colon cancer, Oncological resection, Debridement, Case report

## Abstract

**Introduction and importance:**

Necrotising fasciitis caused by a perforated colon cancer is a very rare occurrence and can be very life threatening needing urgent intervention involving tissue salvage and oncological treatment. There is not enough evidence in the literature regarding management of the same. This case report highlights one such case along with management principles.

**Presenting case:**

We present a 66 year old male with 3 weeks of a progressive right lower quadrant lump and constitutional symptoms. He had a computed tomography scan demonstrating a complex collection in the right anterior abdominal wall, containing multiple locules of gas and air fluid levels near an abnormally thickened hepatic flexure. He was taken for an urgent debridement followed by laparotomy which demonstrated extensive abdominal wall necrotising fasciitis secondary to a perforated hepatic flexure tumour invading into the duodenum. He was given a diverting ileostomy. He had a relook laparotomy the next day for a right hemicolectomy and part of the duodenum resected with a refashioned end ileostomy. He was subsequently managed on the ward for two weeks and then discharged home. He remains well and has been referred to medical oncology for adjuvant chemotherapy.

**Clinical discussion:**

A two step surgical approach was key in this case, first step for source control and the second step focused on an oncological resection.

**Conclusion:**

This case explains the importance of excluding malignant causes of necrotising fasciitis. Perforated cancers can manifest as necrotising fasciitis and management should include timely debridement as well as oncological principles.

## Highlights

• Necrotising fasciitis can be life threatening due to progressive necrosis.
• Perforated cancers are a rare but important cause of necrotising fasciitis.
• Timely debridement is vital as well as finding out the cause for the same.
• Oncological resection is the next vital step after debridement in case of a perforated cancer.

## 1 Introduction

Necrotising fasciitis is a fairly life threatening and aggressive soft tissue infection that needs attention in a timely manner [1]. It can rapidly progress affecting skin, subcutaneous fat, superficial and deep muscular fascia by necrosis. In order to prevent sepsis, multiorgan failure and even death, a quick diagnosis and urgent debridement is important [2].

Management also includes consideration of rare causes such as perforated cancers to be the potential cause. According to Kumar et al., the incidence of necrotising fasciitis secondary to perforated gastrointestinal cancer is only 16% [3]. This case report details one such scenario.

This case report has been produced in keeping with the SCARE criteria [4].

## 2 Case

A 66 year old male presented with abdominal pain and a 3 week history of a right lower quadrant (RLQ) lump that had progressively developed since he attempted to move a large bottle of oil (approximately 15 L). This was associated with 2 weeks of anorexia and a 5 kg weight loss in 2 weeks. He was not clinically obstructed, he had opened his bowels the morning of presentation and was passing flatus. He had no symptoms of nausea or vomiting. He has got no significant medical or surgical history. He was not a diagnosed diabetic and his random blood sugar level was 5 on presentation. He denied any previous medical history including any kidney or liver related conditions. He has never had a colonoscopy in the past and denied any history of bowel cancer or inflammatory bowel disease in the family.

**Fig. 1:**
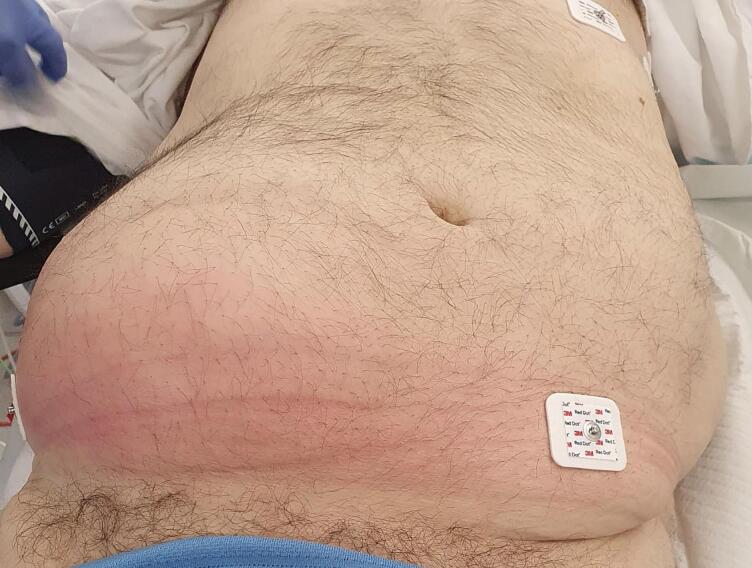
Shows a preoperative image of the patient’s abdomen demonstrating a RLQ lump with distension and overlying erythema.

His vital signs on arrival to the emergency department included the following: Temperature of 37 degree Celsius, Heart rate 75 beats per minute, Blood pressure 123/75 mmHg, Respiratory rate 15, Oxygen saturation 98 % on room air. His abdomen was very distended on examination, with overlying erythema over the right side (Fig. 1). His RLQ was tender over certain parts with a fluctuant mass underneath. There was no crepitus on clinical examination of the abdomen. He had raised inflammatory markers – white cell count (WCC) of 16.4 and C reactive protein of 160. His eGFR on presentation was 80 mL/min (normal) and Creatinine level was 87 (normal range between 60 & 110). His liver function tests were as follows: ALP 190 (slightly elevated, normal range 30–110), GGT 79 (very mild elevation, normal range 5–50), ALT 20 (normal range < 51) AST 29 (normal range < 36).

**Fig. 2:**
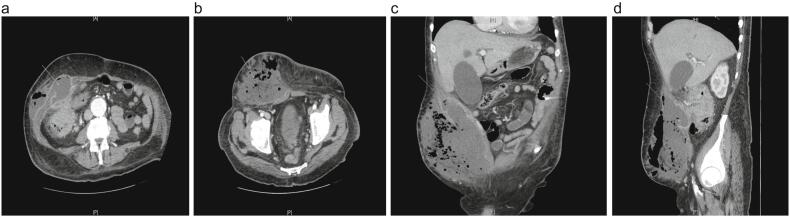
A and B demonstrate an axial view of the complex collection involving the anterior abdominal wall indicated in grey arrows. Panel C in a coronal view and panel D in a sagittal view demonstrate how this collection is in close proximity to a markedly thickened right colon and hepatic flexure in grey arrows.

He underwent a CT Abdomen which demonstrated a complex collection in the right anterior abdominal wall, containing multiple locules of gas and air fluid levels (187 × 91 mm axial dimension and 106 mm craniocaudal dimension) (Fig. 2A and B). This was seen in close proximity to a markedly abnormally thickened right colon and hepatic flexure with significant surrounding oedematous change and fluid (Fig. 2C and D). He was resuscitated with IV fluids and commenced on IV antibiotics.

A bolus of IV Hartmanns was prescribed followed by IV Hartmanns at a maintenance rate of 125 mL/h. Triple antibiotics (empirical treatment for necrotising fasciitis) were charted as per the therapeutic guidelines [5] – IV Meropenem, Vancomycin and Clindamycin.

He underwent an emergency operation during late hours that night, wherein the anterior abdominal wall over the right lower quadrant demonstrated necrotising fasciitis, containing extensive purulent fluid needing drainage, and dead tissue including fascia needing debridement. A transverse incision was made over the right lower quadrant overlying the abscess. Extensive debridement was performed of all necrotic tissue including fascia.

This was then followed by a laparotomy to determine the cause of the necrotising fasciitis. Laparotomy was performed through the initial incision, cut was extended to facilitate adhesiolysis – careful adhesiolysis was done to separate small bowel from the anterior abdominal wall and then further adhesiolysis to separate omentum and hepatic flexure from the anterior abdominal wall. A mass was found at the hepatic flexure which was presumed to be the source of the necrotising fasciitis due to perforation. However, given the mass was quite adherent to the duodenum and due to lack of safety deemed with regards to separating the mass in order to perform a right hemicolectomy, the decision was made to perform a diverting ileostomy and for a relook the next day during day hours.

Ileostomies are normally made on the right side of the abdomen given the location of the terminal ileum, however it was challenging in this case as the anterior abdominal wall was debrided in the right lower quadrant. Hence, an end ileostomy was brought out at a trephine in the left abdomen that was marked preoperatively.

He was sent intubated to Intensive care post operatively, with a view to bring him back to the operating theatre the next day for an oncological resection. He was requiring minimal vasopressor support in Intensive care whilst awaiting the second operation – about 7 mL of Noradrenaline.

**Fig. 3:**
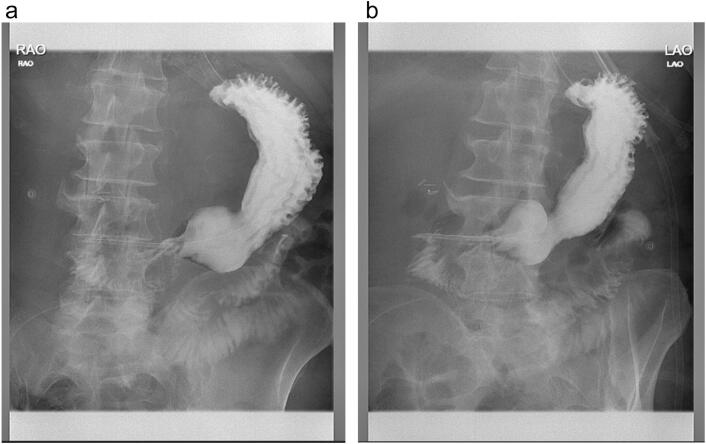
A and B demonstrate a patent duodenal lumen showing contrast entering the jejunum from the stomach on this barium meal study.

During the relook the next day, the mass appeared to be perforating into the duodenum thereby needing a right hemicolectomy with resection of part of duodenum. The 3rd part of the duodenum was involved. Therefore, it was difficult to mobilise the hepatic flexure away from the duodenum as part of the right hemicolectomy. Transverse colon was then resected using a EndoGIA 80 mm stapler. Mesentery was then controlled with a ligasure impact (energy device) followed by stitch ties. Ileocolic pedicle was then taken down. An EndoGIA 60 mm stapler was then used to resect duodenum off hepatic flexure by stapling across the duodenum. A patent lumen was noted post stapling. Transverse colon stump was then brought out via stoma opening – for mucus fistula and end ileostomy was refashioned. His management was then continued with vac dressings and a slow diet upgrade. 2 weeks later, his abdominal wound was eventually closed in the operating theatre. He also had a barium meal which revealed a patent duodenal anastomosis (Fig. 3A and B).

The initial tissue culture showed a heavy growth of mixed coliforms, the histopathology of the abdominal wall was consistent with an abscess – dense neutrophilic abscess in the subcutaneous tissue with necrotic debris. No fungal organisms were seen. There was no evidence of invasive malignancy.

His hepatic flexure histopathology came back positive for high grade adenocarcinoma with mucinous differentiation, loss of MSH-6 → pT4b with tumour perforating into the duodenum. There were 16 lymph nodes in the final specimen but none of them were positive for metastases.

There was positive staining with CK20 and CDX2 and negative staining with CK7. He was then referred to medical oncology for adjuvant chemotherapy who have organised follow up for the patient in the community. He was then discharged home. His chemotherapy was initiated about 4 weeks after the surgery. There were no post operative complications that delayed his treatment.

## 3 Discussion

Perforated cancers that manifest as necrotising fasciitis are fairly rare and do not seem to be very well known in the literature, with only a few reported cases [6]. The incidence of perforated colonic cancer that has been published has been ranging between 3 and 10 [6].

Necrotising fasciitis is more predisposed in patients with diabetes, malignancy, chronic liver and kidney disease as well as a history of alcohol abuse [7]. Signs and symptoms could include presenting septic (fevers, tachycardia, hypotension), shock, erythema, swelling, crepitus, overlying skin changes, subcutaneous gas, pain disproportionate to appearance [7]. Elevated inflammatory markers like WCC and CRP can aid in the diagnosis [7].

Suspicion for necrotising fasciitis warrants an urgent computed tomography (CT) which usually shows a subcutaneous gas and fluid collection [1]. In certain cases, this communicates with a perforated mass [7].

In this case, our patient was very stable and his presentation was not very typical for necrotising fasciitis. He did not have any risk factors like diabetes, immunosuppression or smoking. He presented with a 3 week history of a progressively worsening lump post heavy lifting which was initially suspicious for an incarcerated hernia more than necrotising fasciitis. Therefore, a CT was warranted to further investigate this lump. It is however agreed that if there is a high clinical suspicion for necrotising fasciitis, surgery should not be delayed for imaging [2].

LRINEC (Laboratory Risk Indicator for Necrotizing Fasciitis) score is a diagnostic scoring tool which helps differentiate necrotising fasciitis from other soft tissue infections [8]. It uses six different parameters to do so which include the following: white blood cell (WBC) count, haemoglobin, sodium, glucose, creatinine and C reactive protein [8]. A score of 6 or more is indicative of a “moderate” risk of necrotising fasciitis (50–75 % probability) whereas a score of 8 or more is indicative of a “high” risk (>75 % probability) [8]. However, Fernando et al [8] suggested that a LRINEC score greater or equal to 6 was poorly sensitive and only moderately specific, while a score greater or equal to 8 increased the specificity but the sensitivity was significantly decreased. While it was suggestive that the LRINEC score was associated with poor diagnostic accuracy, the individual scoring elements (for example, WBC or sodium) were more accurate individually [8].

The management of necrotising fasciitis includes Intravenous fluid resuscitation, broad spectrum antibiotics and emergency surgical intervention. Urgent surgical intervention ie debridement down to healthy tissue is still considered gold standard when it comes to management as it aims to stop progression of infection and reduce systemic toxicity [9]. Delay in debridement increases the risk of the already high mortality of 20–30% [1].

In our case given suspicion of malignancy, principals of oncological resection were key. Therefore, a two step approach was useful – the first step helped achieve initial source control and the second step helped focus on the oncological resection, thereby optimising post operative recovery and enabling consideration of adjuvant chemotherapy [10].

Vac dressings have been shown to be very important in the closure of wounds associated with necrotising fasciitis [11]. Vac dressings reduce the use of gauze daily along with toxin absorbance, which thereby results in less pain and less use of narcotics [11]. Vac is also very useful to preserve residual subcutaneous soft tissue, thereby promoting the formation of a better wound bed which can be used in the future for reconstructive surgery [11]. There is evidence that Vac reduces the mortality rate by 27 % compared to simple dressings [11]. However, there is no data in the literature to suggest any significant differences between vac and simple dressings with regards to number of debridements, total length of hospital stay, and complication rates [11]. More studies need to be conducted in order to further evaluate this [11].

There is a controversy about making stomas in such situations **–** The rationale behind choosing an ileostomy and mucus fistula over an ileocolic anastomosis is the fact that there was contamination in the abdomen from a perforated colonic tumour. Given the high risk of anastomotic breakdown in the context of recent contamination and sepsis from the necrotising fasciitis, the decision was made not to perform an anastomosis. Calderillo-Ruiz et al [12] further supports this by suggesting that creation of an ileostomy is a fairly routine procedure performed after colon surgery in cases of threatened anastomosis and/or due to the presence of severe inflammation with the intention of preventing a systemic sepsis due to a possible anastomotic leak. However, they do also highlight that stomas come with short term complications like skin irritation, prolapse, dehydration due to high output and incisional hernias along with long term complications such as kidney injury [12]. However, our patient was referred to a colorectal surgeon for a stoma reversal in the near future.

In conclusion, this case demonstrates an atypical presentation of necrotising fasciitis secondary to a perforated colonic cancer and highlights the importance of considering important rare causes such as perforated cancers to be the potential cause of necrotising fasciitis. This thereby enables us to provide timely management including appropriate surgical intervention. Certain features of patient's history such as development of right lower quadrant lump post heavy lifting and progressive worsening of lump with pain are more suggestive of a hernia over a presentation of necrotising fasciitis. Moreover, the chronicity of his symptoms (ie 3 weeks) makes it less favourable for a necrotising fasciitis. These features thus justify the use of a CT to aid in the diagnosis which was supported by the patient's stable haemodynamic status. This case thus teaches us that sometimes imaging can be useful to diagnose necrotising fasciitis as this changes management – if no imaging was performed and patient was managed as a clinical incarcerated hernia, the operation principles would have been different.

## Funding

There were no sponsors for this case report.

## Ethical approval

This study has been exempted from ethical approval by our institution.

## Consent

Written informed consent was obtained from the patient for publication of this case report and accompanying images. A copy of the written consent is available for review by the Editor-in-Chief of this journal on request.

## Registration of research studies

This case report is not a “First in Man” and therefore does not require any registration.

## Declaration of competing interest

There are no conflicts of interest in the creation of this case report as declared by the authors.

## References

[1] Heidelberg L., Pettke E., Wagner T., Angotti L. (2020). An atypical case of necrotizing fasciitis secondary to perforated cecal cancer. J Surg Case Rep..

[2] Haemers K., Peters R., Wesseling F. (2013). Necrotising fasciitis of the thigh. BMJ Case Rep..

[3] Kumar D., Cortés-Penfield N., El-Haddad H., Musher D. (2018). Bowel perforation resulting in necrotizing soft tissue infection of the abdomen, flank, and lower extremities. Surg. Infect..

[4] Agha R.A., Franchi T., Sohrab C., Mathew G., Kirwan A., Thomas A. (2020). The SCARE 2020 guideline: updating consensus Surgical Case Report (SCARE) guidelines. Int. J. Surg..

[5] Therapeutic Guidelines (2019).

[6] Karam C., Kozman M., Fewtrell M., Ooi K. (2020). Perforated descending colon adenocarcinoma manifesting as necrotizing fasciitis. ANZ J. Surg..

[7] Bahl N., Long A.S., Vemuri A., Jessee T. (2021). A case of necrotizing soft tissue infection secondary to perforated colon cancer. Cereus..

[8] Fernando S. (2019). Necrotizing soft tissue infection: diagnostic accuracy of physical examination, imaging, and LRINEC score: a systematic review and meta-analysis. Ann. Surg..

[9] Evans W., Winters C., Amin E. (2015). Necrotising fasciitis secondary to perforated rectal adenocarcinoma presenting as a thigh swelling. BMJ Case Rep..

[10] Downie E., Bhamidipaty M., Li R., Liang J. (2020). Surgical management of a perforated caecal carcinoma presenting as abdominal wall necrotizing fasciitis. ANZ J. Surg..

[11] Zhang R., Zhang Y., Hou L., Yan C. (2023). Vacuum-assisted closure versus conventional dressing in necrotizing fasciitis: a systematic review and meta-analysis. J. Orthop. Surg. Res..

[12] Calderillo-Ruiz G. (2023). Impact of ileostomy in the adjuvant treatment and outcome of colon cancer. Int. J. Color. Dis..

